# Surface dielectric barrier discharge plasma–treated pork cut parts: bactericidal efficacy and physiochemical characteristics

**DOI:** 10.1016/j.heliyon.2022.e10915

**Published:** 2022-10-03

**Authors:** D. Boonyawan, K. Lamasai, C. Umongno, S. Rattanatabtimtong, L.D. Yu, C. Kuensaen, J. Maitip, P. Thana

**Affiliations:** a Plasma and Beam Physics Research Facility, Chiang Mai University, Chiang Mai, 50200, Thailand; b Doctor of Philosophy Program in Nanoscience and Nanotechnology (International Program/Interdisciplinary), Faculty of Science, Chiang Mai University, Chiang Mai 50200, Thailand; c Department of Animal Science, Faculty of Agriculture at Kamphaeng Saen, Kasetsart University, Kamphaeng Saen Campus, Nakhon Pathom 73140, Thailand; d International College of Digital Innovation, Chiang Mai University, Chiang Mai 50200, Thailand; e Faculty of Science, Energy and Environment, King Mongkut’s University of Technology North Bangkok, Rayong Campus, Rayong 21120, Thailand

**Keywords:** SDBD plasma, Pathogens decontamination, Pork cuts, Meat quality

## Abstract

Maintaining agro-food product safety remains a significant challenge for satisfying local and global consumers in tropical countries. This issue has been growing due to new pathogen strains, low infectious doses, increased virulence, antibiotic resistance, cross-contamination or recontamination of foods, food-contact surfaces, and biocontamination of water within the food production chain. To respond to this situation, we studied the inactivation efficacy of surface dielectric barrier discharge (SDBD) plasma against pathogens on the surface of various pork cut parts, including the loin, hip, belly, liver, and intestine. The SDBD plasma was operated at 0.30 W/cm^2^ in ambient air, with a gap of 5.0 mm between the plasma generator and the sample surface. Up to 96% germicidal efficiency against surface pathogens were observed, showing after 1 min of SDBD plasma exposure. Visualization of reactive species deposition on the treated surface using KI–starch agar gel reagent indicated a non-uniform distribution of the SDBD-generated reactive species on the treated surface. Following the indirect plasma treatment by the SDBD reactor, the overall color of pork cut samples after plasma treatment was significantly different compared with before. However, the surface morphology and structural characterization of the treated pork cut samples were not significantly altered, and residual nitrites and nitrates were lower than the restriction level for safe consumption. The SDBD reactor should be developed further to produce a uniform distribution of reactive species on the meat surface for the improvement of the decontamination effect without undesirable effects on meat quality parameters.

## 1 Introduction

In tropical countries, maintaining agro-food product safety and satisfying local and global consumers have always been significant challenges to the food industry because of the high temperature and humidity of the environment. Along with economic developments and environmental changes, including rapid industrialization–induced global warming, the challenge is ever-increasingly severe due to the emergence of new strains of pathogens, their low infectious doses, increased virulence, resistance to antibiotics, and cross-contamination or recontamination of foods, food-contact surfaces, and microbial contamination of water within the food production chain. Several methods have been developed to respond to these challenges to reduce pathogen residues in fresh food products [1]. Chemical treatments using chlorine, iodine, and hydrogen peroxide are commonly used. However, due to growing public health concerns about the risk of carcinogens, these compounds are banned in many countries [2, 3]. Thermal treatments may ensure a safe level but induce undesirable effects on the nutritional value, freshness, deterioration of color, and sensorial character of fresh products [1, 4]. Thus, effective non-thermal technologies for decontamination as well as preserving the physicochemical, color, texture, nutritional and sensory qualities of fresh food products are required.

Cold plasma processing is a relatively new approach that aims to improve microbiological safety and maintain the sensory attributes of treated agro-foods. To date, intensive studies investigating the decontamination of plant- and animal-based foods have shown considerable potential for the commercial-scale exploitation of cold plasma technologies [5, 6]. Dielectric barrier discharge (DBD) plasma is a promising technology for decontaminating fresh food surfaces [7, 8]. Non-thermal plasma generated by typical DBD reactors in humid air is a source of reactive oxygen and nitrogen species (RONS), especially species with high oxidation potential, including hydroxyl radical (OH), atomic oxygen (O), ozone (O_3_), hydrogen peroxide (H_2_O_2_), hydrogen superoxide (HO_2_), nitric oxide (NO) and nitrogen dioxide (NO_2_) [9,10]. These reactive species play a role in eliminating contamination of fresh food surfaces by inhibiting microorganisms, including harmful bacteria, viruses, and fungi, and pesticide residue removal [7, 11, 12, 13]. Besides, the DBD plasma reactor offers the advantage of convenient operation in different environmental conditions and the capability to treat the large surface area of fresh food efficiently [7]. However, studies on the DBD plasma treatment of fresh food products of pork cuts are rare. Additionally, studies of surface dielectric barrier discharge (SDBD) as a plasma reactor for pork cut treatment are still scarce. This DBD geometry is an indirect treatment and can cover a large target’s surface. The SDBD plasma reactor can be generated RONS using ambient air as plasma gas. Under indirect treatment, a treated sample is placed close to the active plasma region but not in direct contact. The samples were treated by reactive species diffusing from the discharge zone. Because the treated target is not part of an electric circuit of the SDBD, the treated tissue is protected from electrical burns [14]. Thanks to its flexibility and application not limited to small or flat surfaces, SDBD can be used as an alternative tool for bacteria inactivation and shelf-life extension in the meat industry [15]. Thus, because of the lack of research in this area, this work aimed to investigate the potential of SDBD plasma for the decontamination of bacteria on pork cuts and to quantify the impact of plasma treatment on the physicochemical attributes of meat.

## 2 Materials and methods

### 2.1 Sample preparation

Consumer-grade pork cuts were purchased from Tops market (Central Food Retail Co., Ltd.), Chiang Mai, Thailand, one of the highest-class supermarket chains in the country. Pork cuts—loin, hip, belly, liver, intestine, and lard—were packed in new, unused carton boxes (two cuts per box) to avoid causing any external or internal damage and stored in a refrigerator at 4–5 °C until being used (maximum three days of storage). Cuts with a uniform size of 2.0 × 2.0 cm^2^ and appearance without any bruised press and disease symptoms were selected for the experiments.

### 2.2 Surface dielectric barrier discharge (SDBD) reactor

**Figure 1:**
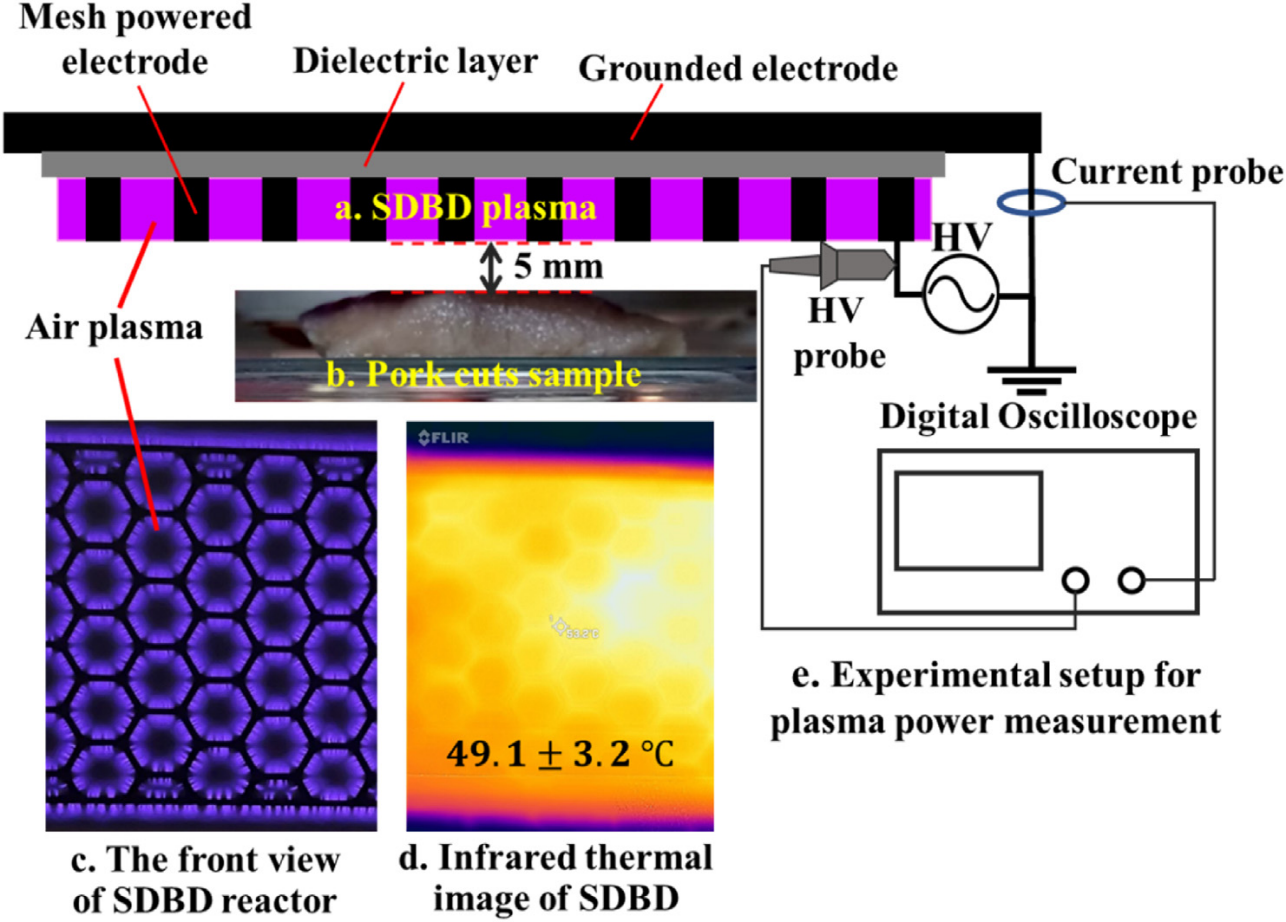
Schematic diagram of the experimental setup for the preparation of SDBD plasma treatment on pork sample: (a) SDBD plasma, (b) pork cut sample, (c) the front view of SDBD reactor, (d) infrared thermal image of SDBD reactor, and (e) experimental setup for plasma power measurement (the white spot due to reflected infrared radiation).

A non-contact SDBD reactor was employed to generate cold air plasma in this study. The experimental configuration is shown in Figure 1. The planar mesh-powered electrode was placed on the lower side of the dielectric sheet, which was made of alumina ceramics with a thickness of 1.0 mm and lateral dimensions of 5.3 cm × 11.3 cm. The aluminum grounded electrode covered the opposite surface of the dielectric. The SDBD operated at atmospheric pressure in humid ambient air (25 °C room temperature, 60% relative humidity). The reactor was driven by a continuous AC high-voltage source of 7.6 kVpp (peak-to-peak) with a repetition rate of 18.2 kHz. The average electrical power dissipated in an AC circle of air plasma discharge was calculated from the expression, $\bar{P}=\left(1/T\right)\int_{0}^{T}V\left(t\right)I\left(t\right)dt$, where *T* is the period of the waveform [16]. The instantaneous voltage across the SDBD reactor *V(t)* and the instantaneous discharge current *I(t)* were measured using a high-voltage (HV) probe (Tektronix P6015A; Tektronix, Inc., United States; 75 MHz bandwidth) and a current probe (PEARSON Current Monitor Model 6585; Pearson Electronics, Inc., United States; 250 MHz bandwidth), respectively. The V–I waveforms were recorded using a digital oscilloscope (Tektronix TDS2014B; Tektronix, Inc., United States; 100 MHz bandwidth). The estimated discharge power was around 18.0 W, and the average surface power density, defined as the discharge power over the SDBD surface area, was 0.30 W/cm^2^. A typical SDBD discharge current waveform consists of a displacement current sinusoidal component and superimposed sharp spikes with a pulse width of 10–50 ns corresponding to the isolated discharge events that appear randomly on the surface of the plasma reactor [17]. Due to the limitation of the used current probe, current spikes were not detected, and the discharge power of this part was excluded from the calculation. Thus, the measured discharge power value would be a lower estimate.

The temperature of the SDBD reactor surface during air plasma discharge was measured by a thermal imaging camera (FLIR ONE; FLIR Systems Inc., United States; with 0.1 °C resolution) after the reactor temperature reached a steady state. The average temperature of the reactor surface was 49.1 ± 3.2 °C during the air plasma discharge.

The SDBD on the pork surface received an indirect treatment, and the treated surface did not make direct contact with the plasma generated at the surface of SDBD. Long-lived reactive species diffusing or drifting from the plasma discharge region play crucial roles in surface modifications, including bacterial inactivation. In all experiments, each pork cut part was treated using SDBD plasma at a distance of 5.0 mm above the sample surface with 1 min of exposure time. Although a shorter distance can result in a better bactericidal effect [18], the distance between the SDBD reactor and the pork surface was fixed at 5.0 mm under the reactor to prevent the treated tissue from electrical and thermal burns. The pork sample was located near the high-voltage electrode, as shown in Figure 1. A shorter distance increases the probability of an electrical arc between the SDBD and the treated surface and can cause damage to pork tissue. Moreover, the gas temperature decreases along with the distance from the SDBD reactor [19]. Thus, a longer distance can reduce the loss of moisture and protect against protein denaturation [20] induced by heat released from the SDBD reactor. It is well known that a longer exposure time can result in better bacterial inactivation; however, a longer exposure time also induces negative effects on pork quality. Jayasena et al. [15] reported that increased SDBD plasma treatment time results in a significant reduction of pork redness, which consumers use as an indicator of the freshness and quality of meat. Moreover, the sensory score for the taste of pork was significantly lower with longer exposure. For the balance between pathogen inactivation and meat quality conservation, 5.0 mm of the reactor–pork sample distance and 1 min of exposure time were used as the treatment parameters in this study.

### 2.3 Optical emission spectra measurement

The SDBD-produced reactive species in the plasma region were observed using optical emission spectroscopy (OES) equipped with an Exemplar LS-Smart CCD spectrometer at a resolution of 1.5 nm. The light emitted from the air plasma volume was obtained through an SMA-905 optical fiber with a diameter of 2.5 mm and configured by a fiberoptic spectrometer for the near UV–VIS range of 200–850 nm wavelength. The integration times were set to 300 ms.

### 2.4 Measuring the concentration of O_3_, H_2_O_2,_ and NO in the gas phase and H_2_O_2_ and NO_3_- deposited in the liquid film over pork cut surfaces

The concentrations of SDBD-produced O_3_, H_2_O_2_, and NO in the gas phase were analyzed at a distance of 5.0 mm below the plasma reactor surface without pork samples. The concentration of gaseous O_3_ was measured using 2B Technologies’ model 106-L ozone monitor (2b Technology Inc., USA) with a resolution of 0.1 ppb, ranging from 0 to 100 ppm. For the measurement of H_2_O_2_ and NO concentrations in the gas phase, two gas detectors were employed: (1) model SKY2000–H2O2 (Shenzhen YuanTe Technology Co., Ltd., China) for measuring H_2_O_2_ concentration with a precision of 0.01 ppm, ranging from 0 to 50 ppm; and (2) model SKY2000–NO (Shenzhen YuanTe Technology Co., Ltd., China) for measuring NO concentration with a precision of 0.01 ppm, ranging from 0 to 100 ppm.

The concentrations of hydrogen peroxide (H_2_O_2_) and nitrate (NO_3_-) deposited in liquid film over plasma-treated pork cut surfaces were determined by using a colorimetric method with two semi-quantitative test strips: (1) 110011-Peroxide Test MQuant® (measuring range 0.5–25 mg/l H₂O₂, Merck KGaA, Darmstadt, Germany) for measuring hydrogen peroxide concentration and (2) 110020-Nitrate Test MQuant® (measuring range 10–500 mg/l NO_3_-, Merck KGaA, Darmstadt, Germany) for measuring nitrate concentration. According to instructions for the use of the products, the determination can be performed not only in liquid samples but also on moist surfaces of freshly cut fruit and vegetables, for example. Thus, these test trips were employed to measure H_2_O_2_ and NO_3_- in liquid film over pork cut surfaces. The reaction zone of the test strip makes contact with the moist surface for 1–10 s and after 1 min compares with the color scale.

### 2.5 Visualization of reactive species deposition on the treated surface

The spatial distribution of SDBD-generated RONS on the treated surface was visualized using the potassium iodide (KI)–starch method [21]. To prepare a gel reagent, the commercial-grade agar powder was mixed with DI water for 0.5% w/v concentration. The KI for analysis powder (Sigma-Aldrich, Germany) and starch-soluble GR for analysis (Merck KGaA, Germany) were dissolved for each 0.5% w/v in the agar solution. The solution was placed in a Petri dish (8.79 cm in diameter) and heated by the autoclave at a temperature of 121 °C. Finally, the solution became a gel at room temperature. The SDBD reactor was located 5.0 mm above the surface of the KI–starch agar gel reagent during plasma treatment.

### 2.6 Surface morphology and color observation

The surface morphology of the pork cut samples was observed using a 500X portable USB digital microscope (with a 2.4-inch HD screen-integrated stand). The color of the pork cut sample surface was evaluated by a colorimeter (CM-2600d Spectrophotometers Konica-Minolta Sensing, Osaka, Japan) to determine the L∗-, *a∗-,* and *b∗-*values of the CIELAB color space. The *L∗*-axis, *a∗*-plane, and *b∗*-plane represent the degree of brightness, greenness/redness, and blueness/yellowness of the sample. The measurements were performed at 10 different sites per sample. The overall color change after SDBD plasma treatments was indicated by *ΔE∗* calculated from the following equation:

(1) $$\Delta E^{*}=\sqrt{{\left(L_{i}^{*}-L_{f}^{*}\right)}^{2}+{\left(a_{i}^{*}-a_{f}^{*}\right)}^{2}+{\left(b_{i}^{*}-b_{f}^{*}\right)}^{2}}$$

Subscripts *i* and *f* represent the values before and after SDBD plasma treatment, respectively [22, 23].

### 2.7 Analysis of bacteria inactivation efficacy

The decontamination efficacy of the SDBD plasma treatment against total aerobic bacteria and three pathogens—*Staphylococcus aureus* (*S. aureus*), *Escherichia coli* (*E. coli*), and Salmonella spp.—on different pork part surfaces was studied using a swab test by conventional swabbing procedures. In brief, a sterile cotton swab with an applicator stick was swabbed on the surface and then released into the sterile saline solution (0.85%), followed by rigorous mixing and direct or diluted plating. The protocol for the swab test was described in [24]. The proper dilutions of the mixture were then spread on tryptic soy agar (TSA) for total aerobic bacteria enumeration, followed by incubation at 37 °C for 24 h.

After incubation, the number of colonies was counted. For the bacteria enumeration of the three pathogens, the pork samples were sent to the Central Laboratory (Thailand) Co., Ltd. for the second test’s professional measurements of the remaining bacteria on the treated surface. The *E. coli* and *S. aureus* enumerations were performed following the FDA’s Bacteriological Analytical Manual (BAM), Chapters 4 and 12, respectively. Meanwhile, a method based on ISO 6579-1:2017 was used to detect Salmonella spp.

The results expressed in colonies forming units (CFU) per 4-cm^2^ of the sample area were calculated, and the mean values were shown in the bacterial inactivation curves. The results from the two sources were then compared and further analyzed. The germicidal efficiency was determined as follows:

(2) $$\text{Germicidal ​efficiency}=\left(N_{0}-N_{t}\right)/N_{t}\times 100\%$$

where *N*_0_ and *N*_t_ are the numbers of colony-forming units of the untreated and SDBD plasma-treated samples, respectively.

### 2.8 ATR-FTIR spectroscopy analysis

The structural characterization of pork cut samples was examined using a qualitative analysis of Attenuated Total Reflection–Fourier Transform Infrared (ATR-FTIR) spectroscopy. FTIR spectra were obtained using a Nicolet 6700 FTIR spectrometer (Thermo Fisher Scientific, Waltham, MA, USA). The pork cut samples with a size of 1.0 × 1.0 cm^2^ were placed in direct contact with ZnSe 45° ATR (attenuated total reflectance) crystal on the spectrometer. The penetration depth of the infrared (IR) beam into the sample is 0.66 μm. FTIR spectra were collected from 64 scans at a resolution of 4 cm^−1^ over the wavenumber range of 4,000 to 400 cm^−1^ operated at 25 °C room temperature.

### 2.9 Statistical analysis

The data were analyzed using IBM SPSS Statistics 28 software. Statistical analysis was performed using a one-way analysis of variance (ANOVA). The significant difference in mean values between SDBD-treated and untreated samples was determined by Dunnett’s pairwise comparison test. Data with p-values smaller than 0.05 (p < 0.05) were considered statistically significant. The data were reported as mean values, and standard deviations (SDs) from three replicates in each experiment were reported.

## 3 Results and discussion

### 3.1 Optical emission of SDBD plasma

**Figure 2:**
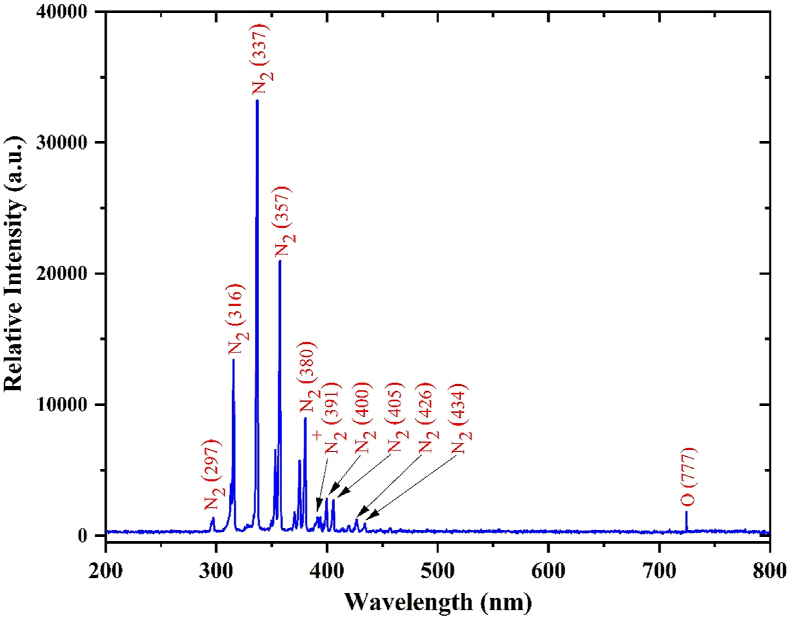
Optical emission spectrum of air plasma generated by the SDBD plasma reactor.

The emission spectrum from the SDBD-produced air plasma measured in the wavelength range of 200–800 nm is shown in Figure 2. The spectra clearly show the dominant emission band of N_2_ second positive system $\left(C^{3}\Pi_{u}\to B^{3}\Pi_{g}\right)$ with a weak NO_2_^+^ first negative band $\left(B^{2}\Sigma_{u}^{+}\to X^{2}\Sigma_{g}^{+}\right)$ commonly found in air plasma or the discharge of inert gas in the air [25]. In discharge processes, most electrical energy dissipated in air DBD plasma is consumed in the excitation, ionization, and dissociation processes of N_2_ molecules [26]. In addition, an emission line of atomic oxygen (O) at 777.4 nm was observed. These reactive species can further react with each other or with surrounding molecules to produce long-lived RONS, such as nitric oxide (NO), ozone (O_3_), HO_2_, nitrous acid (HNO_2_), nitric acid (HNO_3_), and hydrogen peroxide (H_2_O_2_) outside of the plasma discharge region [27, 28]. The RONS generated in core air plasma at the surface of SDBD are delivered through an air gap to the target surface by diffusion and chemical processes within the plasma afterglow [29].

### 3.2 Concentration of H_2_O_2_, NO, and O_3_ in the gas phase and H_2_O_2_ and NO_3_- deposited in liquid film over pork cut surfaces

The concentrations of H_2_O_2_, NO, and O_3_ in the gas phase measured at a distance of 5.0 mm under the SDBD reactor surface without pork samples were 0.0, 0.1, and 6.5 ppm, respectively. This result implied that the concentration of SDBD-generated H_2_O_2_ in the gas phase was less than 0.01 ppm (the resolution of the H_2_O_2_ gas detector). Meanwhile, the density of hydrogen peroxide (H_2_O_2_) and nitrate (NO_3_-) deposited in liquid film over the plasma-treated surfaces can be detected. The concentrations of H_2_O_2_ and NO_3_- in liquid film on the pork loin surface after 1 min of SDBD plasma treatment were 2 ppm and 25 ppm, respectively. The density of H_2_O_2_ and NO_3_- measured on other plasma-treated pork cuts (hip, belly, liver, and intestine) were approximately equal in the case of pork loin. It was noticed that plasma-generated H_2_O_2_ cannot be detected in the gas phase but can be found in the liquid phase. Typically, pork cut tissues are covered with thin liquid film. These H_2_O_2_ generated in plasma-treated liquid could be formed from the reaction between plasma-produced O_3_ and water molecules on the treated surface ($O_{3}+H_{2}O\to H_{2}O_{2}+O_{2}$) [30]. Furthermore, the reactive species in the gas phase can react with water molecules on the treated surface to form new RONS in the liquid, such as OH, H_2_O_2_, HO_2_, nitrite (NO_23_-), NO_3_-, and peroxynitrite (OONO^−^) [29]. For example, H_2_O_2_ and NO_2_- (generated from NO) found in this study can react with each other and form peroxynitrous acid (ONOOH) [31]. It is commonly known that bacterial inactivation in the liquid phase is not a result of individual species but a mixture of reactions induced by several radicals. It is a synergistic contribution of various necessary RONS, such as OH, H_2_O_2_, nitrate (NO_3_-), nitrite (NO_2_-), O_3_, and peroxynitrite [28, 32].

### 3.3 Visualization of reactive species deposition on the treated surface

**Figure 3:**
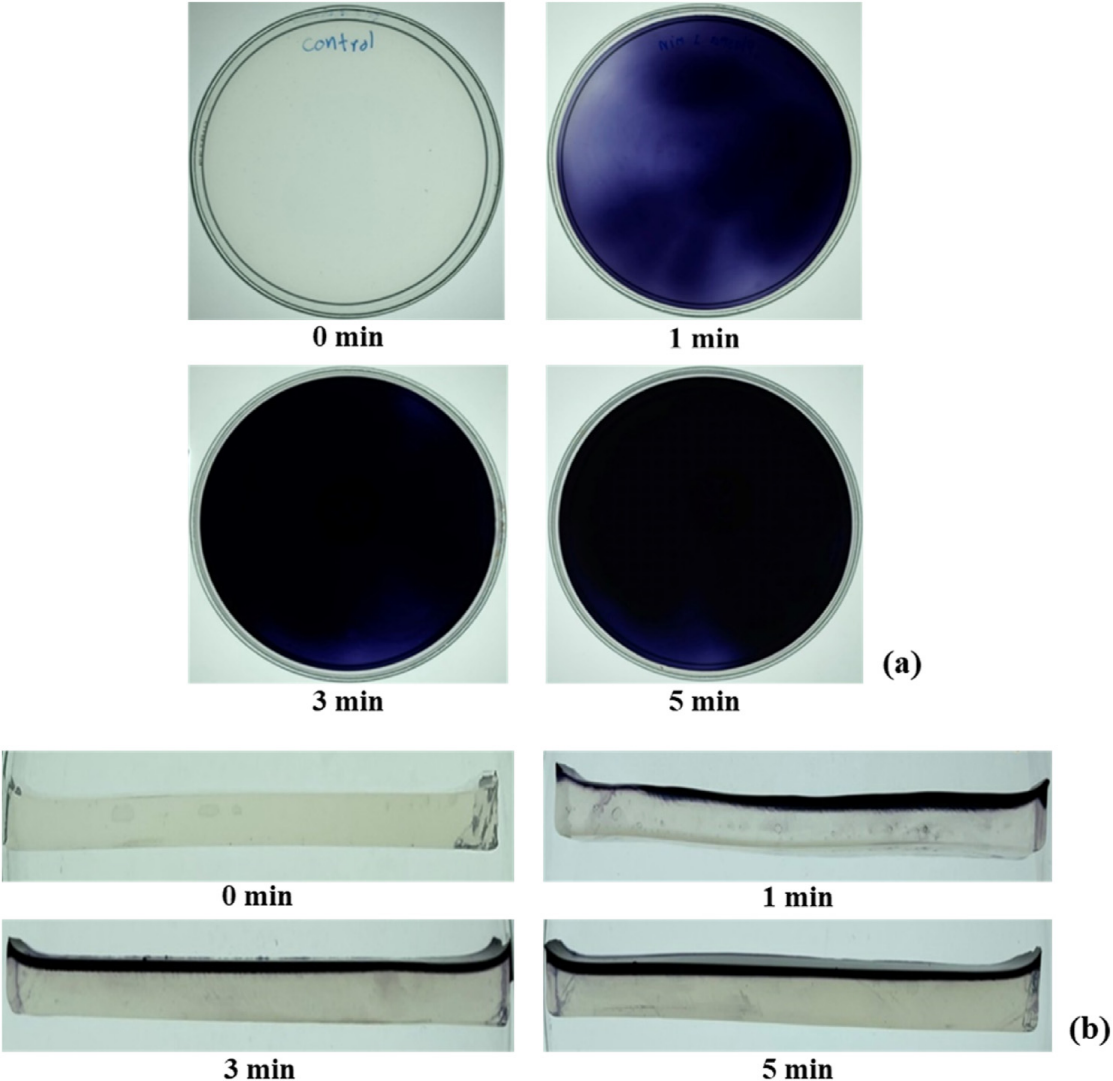
KI–starch agar gel reagent after SDBD plasma treatment with various exposure times: (a) Spatial distribution of RONS deposit on the plasma-treated surface (b) The depth of penetration profiles of RONS into the gel reagent.

The spatial distribution of reactive species at the target’s surface is a crucial factor determining the effects of SDBD on the surface, including pathogen inactivation efficacy. The spatial distribution of SDBD plasma-generated RONS deposited on the treated surface was studied using the KI–starch agar gel reagent. This visualized method is based on a conventional iodine-starch color reaction. The abundance of iodide (${\text{I}}^{-}$) deposited in the gel reagent can be oxidized to iodine (I_2_) by the presence of plasma-produced RONS, which have higher oxidation potential (E^0^) than I_2_ (E^0^ = 0.54 V). The oxidation potential of important plasma-generated reactive species is shown in Table 1. The I_2_ reacts with another ${\text{I}}^{-}$ to form triiodide (${\text{I}}_{3}^{-}$). Triiodide then forms complexes with starch, and the color turns blue [33]. Thanks to the most reactive species generated by plasma having a higher oxidation potential than I_2_, the KI–starch gel method can visualize the overall distribution of RONS in a single measurement. After 1 min of SDBD plasma treatment, most of 60.7-cm^2^ of gel surface had a colored area, as shown in Figure 3(a). This result indicated that reactive species generated by SDBD plasma could be deposited on most of the area of the treated surface. The darkened color area showed a high density of reactive species deposited in the area. The non-uniform color distribution occurred due to the non-homogeneous plasma discharge on the SDBD surface. However, increasing the exposure time led to a homogeneous distribution of RONS covering the most treated areas. The non-uniform release of the SDBD reactor may affect the efficacy of pathogen inactivation on pork cut products. Therefore, further studies are required to improve an efficient SDBD plasma reactor that can be applied effectively in the food industry.

**Table 1:** The oxidation potential of important plasma-generated reactive species [10].

Species	Oxidation potential, E^0^ (V)
Hydroxyl Radical (OH)	2.86
Atomic Oxygen (O)	2.42
Peroxynitrous Acid/Peroxynitrite ($\text{ONOOH}/{\text{ONOO}}^{-})$	2.10
Ozone (${\text{O}}_{3}$)	2.07
Hydrogen Peroxide (${\text{H}}_{2}{\text{O}}_{2}$)	1.78
Hydrogen Superoxide (${\text{HO}}_{2}$)	1.50
Superoxide (${\text{O}}_{2}^{-}$)	1.00
Nitrate (${\text{NO}}_{3}^{-}$)	0.96
Nitric Oxide (NO)	0.90
Nitrogen Dioxide (${\text{NO}}_{2}$)	0.90

The penetration capacity of plasma-generated RONS is an essential factor in determining the biological effect of plasma-generated RONS on the deep tissue of the treated sample and the accumulation of residual RONS [34]. To study the depth of SDBD plasma RONS penetration into the treated surface, the treated KI–starch agar gel reagents were vertically cut in half, as shown in Figure 3(b). The cross-section of the gels showed the depth-direction profiles of RONS deposited on the treated gel reagents. The penetration profile was non-uniform after 1 min of treatment. However, the penetration depth remained constant, with uniform penetration after 3 min of treatment. The maximum penetration depth of RONS into KI–starch agar gel was around 1.0 mm with 5 min of treatment time. Duan et al. performed a study on the penetration depth of RONS generated by a plasma jet through actual biological tissue [31]. They studied the penetration depth of plasma jet–produced ROS (OH, H_2_O_2_, and O_3_) and RNS (NO_2_- and NO_3_-) through pig muscle tissues. There are two interesting results in this study. First, they found different behaviors between plasma-produced ROS and RNS during penetration through pig tissue. When the thickness of the pig muscle tissue is 0.5 mm and the plasma exposure time is 10 min or 15 min, more than 80% of the RNS, including NO_2_- and NO_3_- generated by the plasma, can penetrate through the tissue. On the other hand, less than 5% of ROS, including OH, H_2_O_2_, and O_3_, can penetrate tissue with the same thickness. This indicates that the plasma-produced ROS are consumed more significantly by the tissue than by RNS. This phenomenon is due to the different reaction mechanisms of ROS and RNS with the organism in the biological tissue. The maximum depth of the plasma jet–generated RONS penetrating the pork tissue was 1.5 mm in the study. Another interesting result was that the penetration depths were proportional to the plasma treatment time. In addition, the penetration depths of plasma-generated RONS were also depended on the RONS species density in the gas phase and the structural characteristics of the treated samples [34].

Thanks to their recognized antimicrobial effects against pathogenic bacteria, small amounts of nitrite (NO_2_-) and nitrate (NO_3_-) are authorized as food additives in meat products as a preservative [35]. However, nitrite can be a precursor of nitrosamines, many of which are known to be carcinogenic [36]. Thus, further studies are needed to optimize the plasma treatment doses, which connect the various plasma parameters, such as electric power, the density of each plasma-generated species, and the plasma treatment time and moisture content of the treated surface [37]. For this optimized treatment dose, effective antimicrobial effects would be obtained with an acceptable amount of residual nitrite and nitrate in plasma-treated pork cuts within standards such as those of the European Union under Commission Regulation (EU) No 1129/2011 and the Joint FAO/WHO Expert Committee on Food Additives (JECFA) [35].

### 3.4 Surface morphology and color measurement

**Figure 4:**
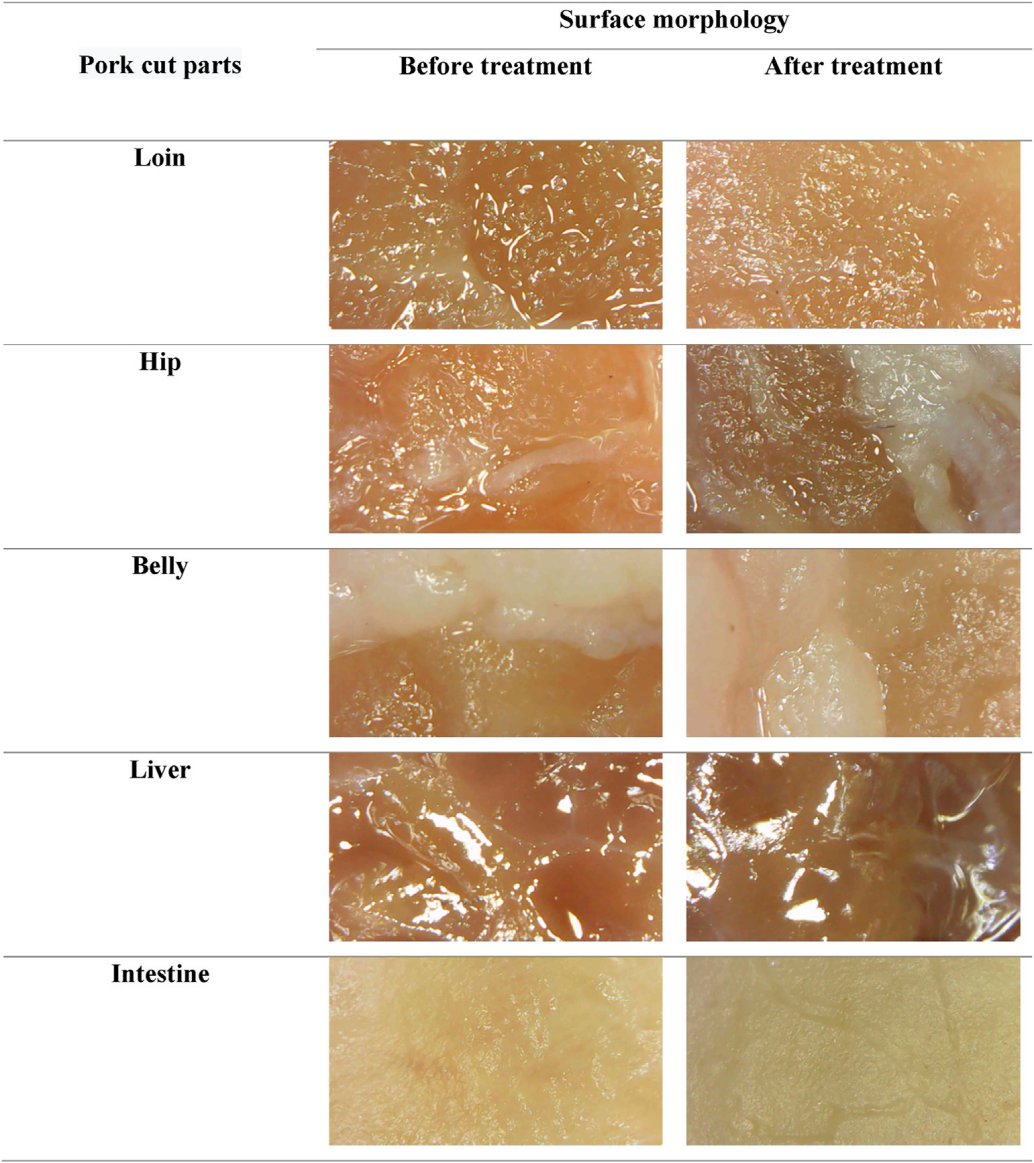
The surface morphology of pork cuts before and after SDBD plasma treatment.

**Figure 5:**
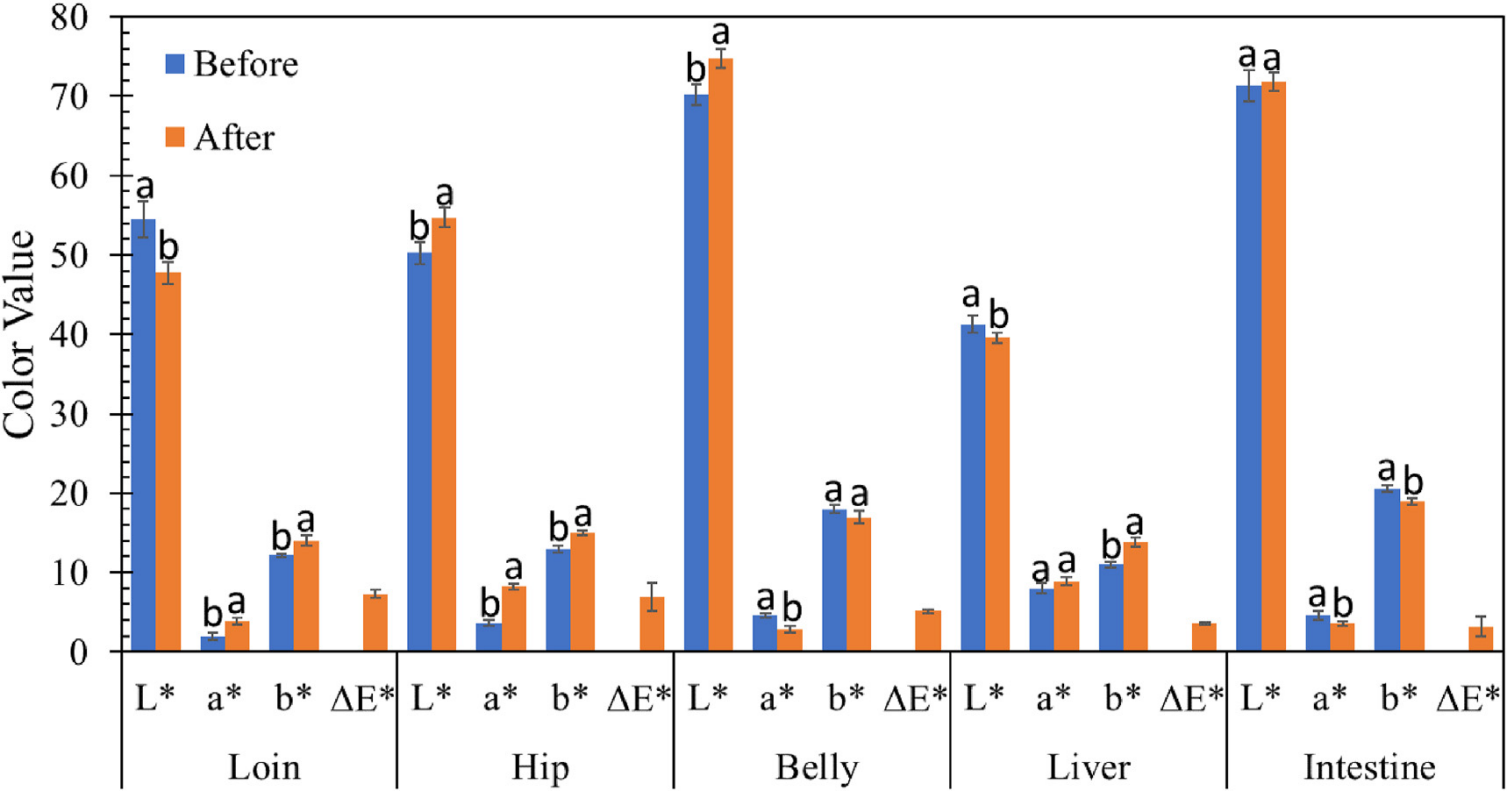
CIE L∗–a∗–b∗ values and total color difference (ΔE∗) values of pork cuts before and after the SDBD plasma treatment. Different small letters (a, b) indicate significant (p < 0.05) differences in color values before and after SDBD plasma treatment. Δ*E∗* value.

Surface morphology and color are critical quality attributes and are also a significant factor affecting consumers’ assessment of pork quality [4, 15]. Figure 4 shows the surface morphology of the pork cut samples before and after 1 min of plasma treatment. There was no visible electrical burn or thermal damage [38] on the SDBD-treated pork cut samples. The color value changes in pork cuts treated with SDBD plasma are shown in Figure 5. Many factors are involved in the discoloration of pork cuts. The increase in lightness (*L∗*) might result from cell perforation caused by plasma treatment [39]. Meanwhile, the decrease in surface moisture may have contributed to the decrease in lightness [4, 40]. Water evaporation from the treated surface is induced by heat released from the air plasma discharge processes. Lightness also increases with temperature due to protein denaturation [41]. Myoglobin pigments, including deoxymyoglobin, oxymyoglobin, and metmyoglobin, play a crucial role in the color of meat and are closely related to its redness. The redness of meat might be increased because plasma-generated nitric oxide (NO) reacts with deoxymyoglobin or metmyoglobin to form bright-red nitrosylmyoglobin or dark-red nitrosometmyoglobin, respectively [42]. A significant increase in meat redness (*a∗-*value) after treatment with either plasma or plasma-treated water was reported in various studies [43, 44, 45]. Conversely, red oxymyoglobin and purple deoxymyoglobin can be oxidized by plasma-produced RONS to brown metmyoglobin, and the color of meat can be altered to lower redness and higher yellowness [46, 47]. Plasma-produced hydrogen peroxide can react with myoglobin and form choleglobin, causing the meat to appear greener (decrease *a∗*-value) [4]. Moreover, green discoloration probably occurs because SDBD-produced nitrite (NO_2_-) reacts with myoglobin to form green nitrimyoglobin [48]. Lipid oxidation induced by plasma-generated RONS can be a reason for a decrease in redness (*a∗*) [42]. Plasma-generated reactive species could exacerbate lipid oxidation in the pork sample and change the b∗-values [39]. According to Eq. (1), the overall color change (Δ*E∗*) values of most SDBD-treated pork cut samples were in the range from 3.2 to 7.5. These differences in perceivable color, with Δ*E∗* > 3.0, indicated that the colors of pork cut samples after SDBD plasma treatment were very distinct from before [23]. Further studies are needed to optimize the adverse effects of SDBD treatment parameters on raw meat color.

### 3.5 Effect of SDBD plasma treatment on decontamination

**Figure 6:**
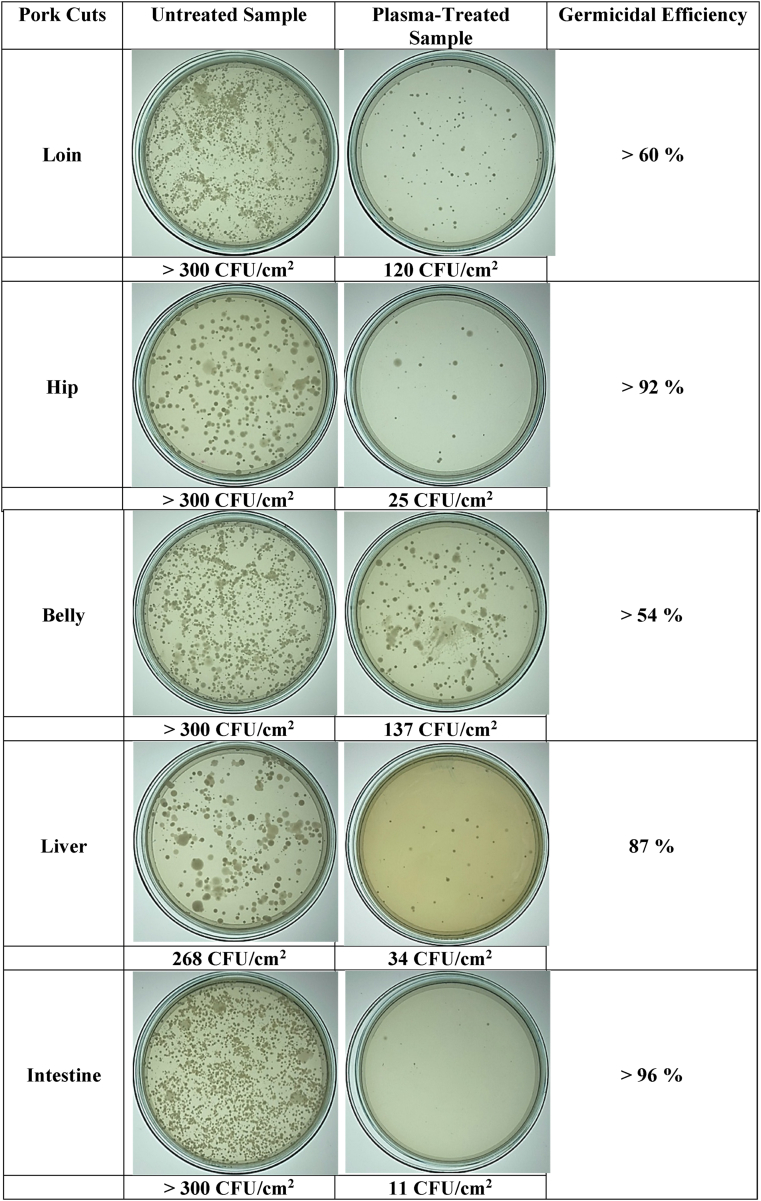
The effect of SDBD plasma treatment on decontamination of the different parts of pork cuts against total bacteria after exposure time of 1 min. The total aerobic bacteria count of the different parts of the pork cut surface between untreated and plasma-treated groups on tryptic soy agar (TSA) was expressed in CFU/cm^2^, and the germicidal efficiency was expressed as a percentage.

**Figure 7:**
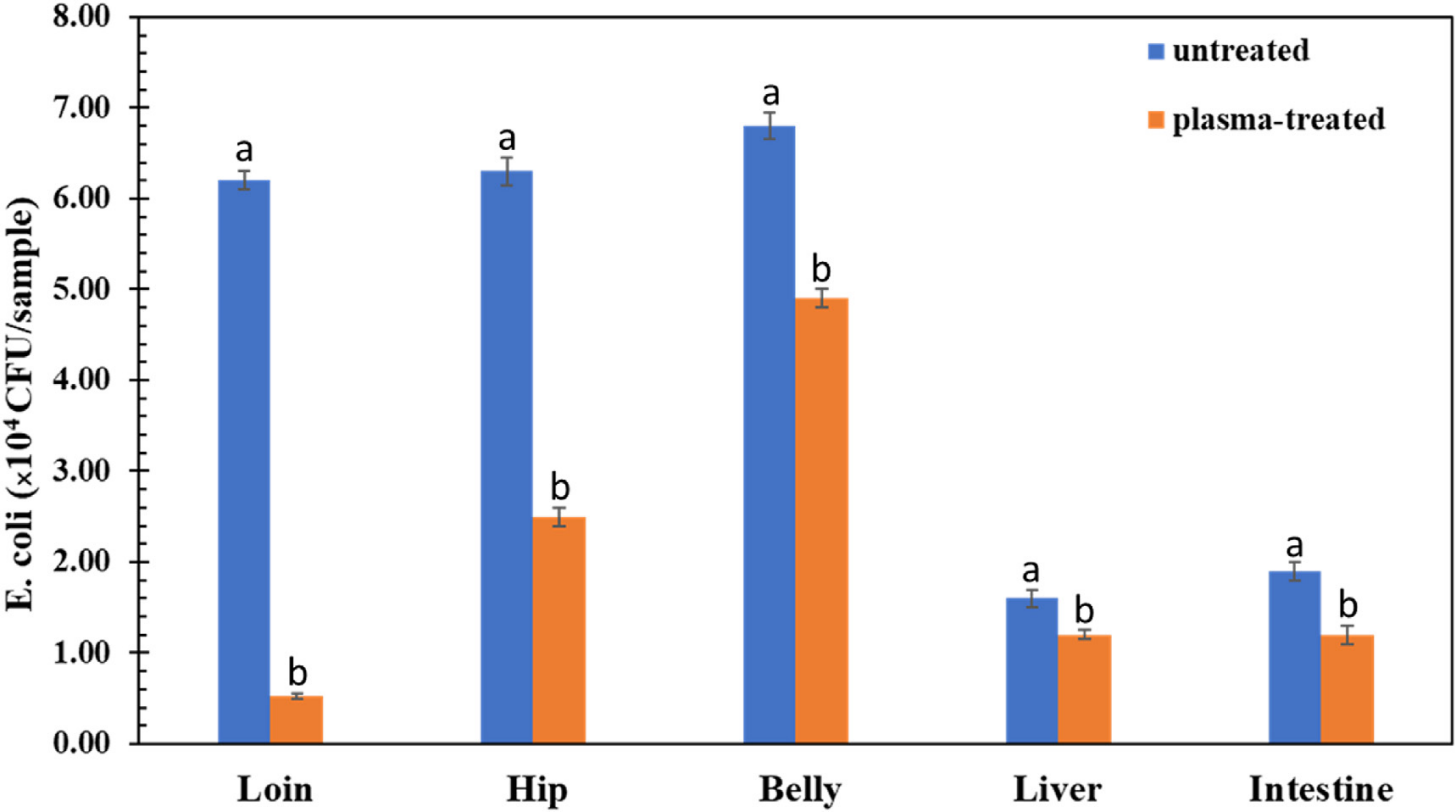
Results of SDBD plasma treatment effect on bacteria inactivation of pork parts obtained from professional evaluation. Different small letters (a,b) indicate significant (p < 0.05) differences of *E. coli* in SDBD-treated and untreated samples.

The effects of SDBD plasma treatment on the decontamination of the total aerobic bacteria on pork cut surfaces are shown in Figure 6. The results showed an apparent inactivation of the total aerobic bacteria on pork cuts compared with the untreated and the 1-min treatment with SDBD plasma. The germicidal efficiency evaluated using Eq. (2) of SDBD plasma treatment on pork cut parts against total aerobic bacteria after 1 min of treatment time was between 54% and 96%. Another evaluation of the remaining three pathogenic bacteria (*E. coli*, *S. aureus*, and *Salmonella* app.) after treatment with SDBD plasma was obtained from professional measurements of the Central Laboratory (Thailand) Co., Ltd. First, the results indicated that the most pathogenic bacteria on pork cut surfaces in this study were *E. coli, while S. aureus* and *Salmonella spp.* were negligible. The number of *E. coli* on the surface of various pork cut parts, including the loin, hip, belly, liver, and intestine, compared between untreated and SDBD plasma–treated samples, are shown in Figure 7. The germicidal efficiency of the SDBD plasma treatment on the loin, hip, belly, liver, and intestine was 91%, 62%, 28%, 25%, and 36%, respectively. These results indicated that the ability of SDBD plasma to reduce the number of bacteria was significantly reduced after plasma treatment. Consequently, SDBD plasma showed the potential to inhibit aerobic bacteria in pork cuts. The lower reduction levels may still be sufficient to reduce pathogenic bacteria, such as *E. coli*, in raw poultry and render it safer for consumers [49]. The SDBD air plasma produced reactive species responsible for oxidation reaction that affects the cell wall and cell membrane of bacteria that are destroyed and cytoplasm that is released to cause the death of the bacteria [4, 15, 50]. The reasons for the different bacteria inactivation efficiency of the SDBD plasma on various pork cut samples might be the non-uniform discharge of SDBD reactor, which is discussed in Section 3.3, the different surface properties, such as moisture content, nutrient content, and surface topology, as well as the plasma treatment dose [37, 51]. Therefore, SDBD plasma treatment conditions may vary in actual applications.

### 3.6 ATR-FTIR spectroscopy analysis

**Figure 8:**
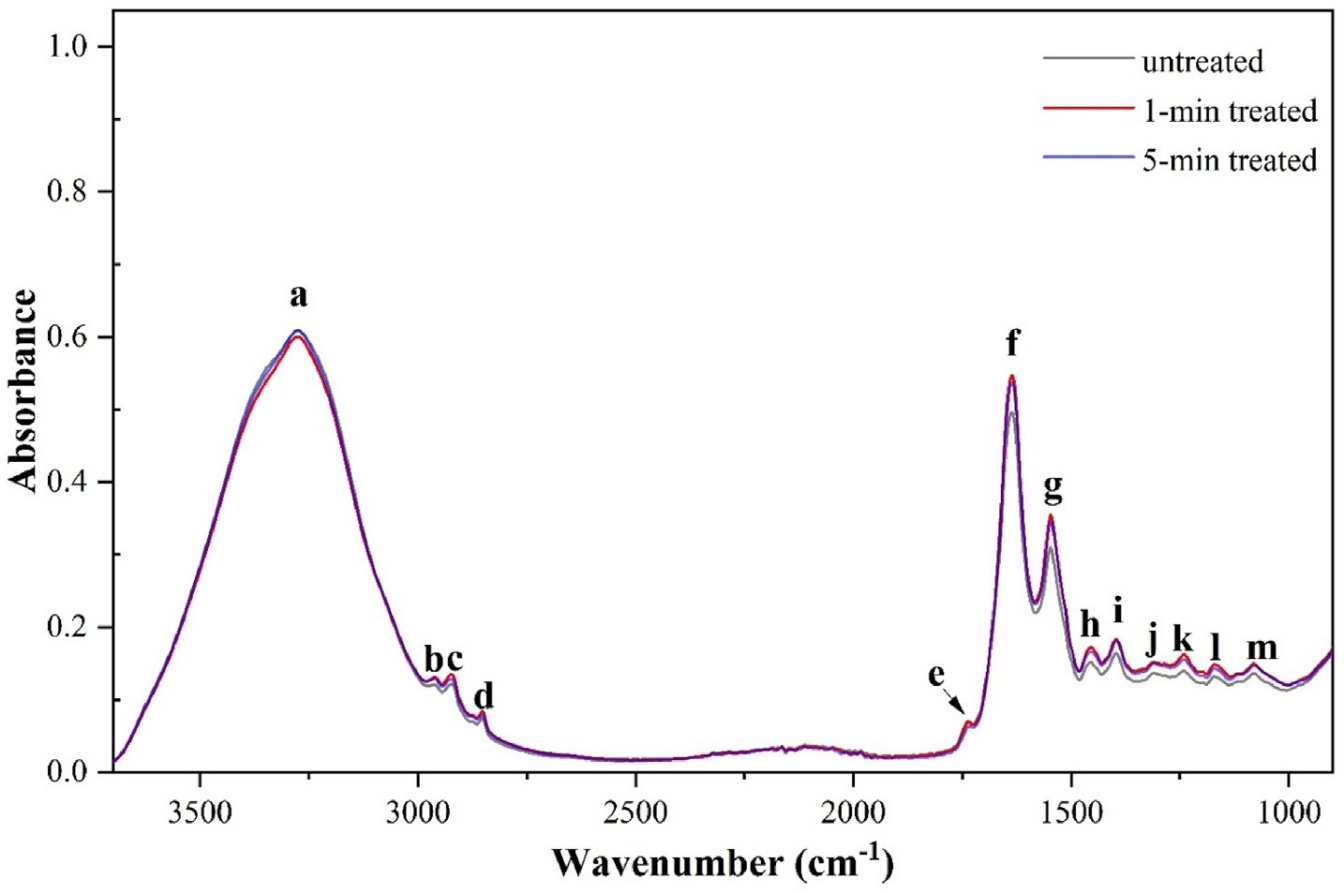
FTIR spectra of control and SDBD plasma–treated pork loin.

FTIR absorption spectra of the SDBD plasma–treated pork loin compared with untreated samples are shown in Figure 8. Specific functional groups in the convoluted infrared absorption spectra were identified using [52, 53]. The characteristic absorption band of proteins occurred at approximately 2386, 1645, and 1545 cm^−1^ (band a, f, and g). The broad and robust absorption peak around 3286 cm^−1^ is due to the N–H stretching vibration of the amide of proteins and the O–H bond of water and polysaccharides. The bands at 1645 and 1545 cm^−1^ are attributed to amide I (C=O stretching, N–H bending, and C–N stretching) and amide II (N–H bending and C–N stretching). Meanwhile, the strong absorption bands at around 2917, 2850, and 1744 cm^−1^ (band c, d, and e) are the characteristic of lipids. The absorptions around 2917 and 2850 cm^−1^ are caused by asymmetric and symmetric stretching vibration CH_2_, respectively. The stretching vibration of C=O carbonyl–related cholesterol esters and triglyceride esters occurs around 1744 cm^−1^. Other infrared absorptions were also observed. The small band b at approximately 2954 cm^−1^ (band b) is the absorption of CH_3_ asymmetric stretching of lipids and proteins. Band h, 1454 cm^−1^, is attributed to the C–O–H bending modes of methyl groups related to proteins and lipids. The peak 1390 cm^−1^ (band i) is due to the vibration of COO^−^ symmetric stretching of fatty acids. The absorption at 1311 cm^−1^ (band j) occurs due to the vibration of amide III (C–N stretching, N–H bending, C=O stretching, and O=C–N bending). The asymmetric stretching vibration of PO_2_^-^-related nucleic acids (mainly), phospholipids, and phosphorylated proteins occur at 1241 cm^−1^ (band k). The 1172-cm^−1^ peak (band l) is the absorption band of the CO stretching vibration of the C–OH groups of serine, threonine, tyrosine residues, and the C–O stretching of carbohydrates. Finally, the absorption around 1097 cm^−1^ (band m) is due to PO_2_^-^ symmetric stretching, C–O stretching, C–H deformation–related nucleic acids, phospholipids, polysaccharides (glycogen), fatty acids, and esters of lipids.

The spectra in Figure 8 showed that the infrared absorptions of the SDBD plasma–treated pork samples were not significantly different from the untreated sample. The absorbance of the broad range around 3286 cm^−1^ with related O–H groups of hydrogen peroxide was not a significant change. In addition, the broadband centered at 1348 cm^−1^ is due to H–O–O-bending vibrations of H_2_O_2_ molecules [52] disappearing in the spectra of SDBD plasma–treated samples. These absences of H_2_O_2_ absorption spectra implied no significant amount of H_2_O_2_ residual in the pork tissue. Moreover, the infrared absorptions associated with NO_2_- (1245 cm^−1^) and NO_3_- (1401 cm^−1^ and 1350 cm^−1^) [53] were not found in the spectra of SDBD-treated samples. This result indicated that SDBD plasma–generated nitrite and nitrate accumulation in the tissue bulk of the plasma-treated pork cut samples were insignificant.

## 4 Conclusion

In summary, this study demonstrates that indirect plasma treatment with the SDBD reactor has the potential to decontaminate total aerobic bacteria on pork cut surfaces, especially *E. coli.* Deposition on the treated surface of long-lived reactive species diffusing from the plasma discharge region was visualized using a KI–starch agar gel reagent. The visualization indicated the non-uniform spatial distribution of reaction species on the treated surface. To improve the decontaminant effect on fresh pork cuts, the present SDBD reactor must be developed further to generate a homogeneous distribution of reactive species on the treated surface. In addition, future studies will focus on minimizing the undesirable effects of SDBD-generated RONS on meat quality parameters. The toxicity of plasma-treated fresh meat should also be further investigated to enable the use of cold plasma technology in the meat industry.

## Declarations

### Author contribution statement

D. Boonyawan: Conceived and designed the experiments; Analyzed and interpreted the data; Contributed reagents, materials, analysis tools or data; Wrote the paper.

K. Lamasai: Performed the experiments; Wrote the paper.

C. Umongno: Performed the experiments.

S. Rattanatabtimtong: Conceived and designed the experiments; Contributed reagents, materials, analysis tools or data.

L.D. Yu; C. Kuensaen: Analyzed and interpreted the data.

J. Maitip: Analyzed and interpreted the data; Wrote the paper.

P. Thana: Conceived and designed the experiments; Performed the experiments; Analyzed and interpreted the data; Contributed reagents, materials, analysis tools or data; Wrote the paper.

### Funding statement

D. Boonyawan was supported by Agriculture Development Agency (ARDA), Thailand (NRCT 5715/2018).

### Data availability statement

Data will be made available on request.

### Declaration of interest’s statement

The authors declare no conflict of interest.

### Additional information

No additional information is available for this paper.
